# CogTale: an online platform for the evaluation, synthesis, and dissemination of evidence from cognitive interventions studies

**DOI:** 10.1186/s13643-021-01787-2

**Published:** 2021-08-24

**Authors:** Julieta Sabates, Sylvie Belleville, Mary Castellani, Tzvi Dwolatzky, Benjamin M. Hampstead, Amit Lampit, Sharon Simon, Kaarin Anstey, Belinda Goodenough, Serafino Mancuso, Davis Marques, Richard Sinnott, Alex Bahar-Fuchs

**Affiliations:** 1 Academic, Unit for Psychiatry of Old Age, Department of Psychiatry, University of Melbourne, Melbourne, Australia; 2 Psychology Department, Université de Montréal, Montreal, Canada; 3 Research Center, Institut Universitaire de Gériatrie de Montréal, Montreal, Canada; 4 Bruce and Ruth Rappaport Faculty of Medicine, Technion-Israel Institute of Technology, Haifa, Israel; 5 Geriatric Unit, Rambam Health Care Campus, Haifa, Israel; 6 Research Program on Cognition and Neuromodulation Based Interventions, Department of Psychiatry, University of Michigan, Ann Arbor, MI USA; 7 Mental Health Service, VA Ann Arbor Healthcare System, Ann Arbor, MI USA; 8 Department of Neurology, Charité University Hospital, Berlin, Germany; 9 Cognitive Neuroscience Division, Department of Neurology, Columbia University, New York, NY USA; 10 Old Age Research Group (PROTER), Department of Psychiatry, São Paulo Medical School, University of São Paulo, São Paulo, Brazil; 11 UNSW Ageing Futures Institute, University of New South Wales, New South Wales, Australia; 12 UNSW Neuroscience Research Australia, Sydney, Australia; 13 Dementia Training Australia, Faculty of Science Medicine and Health, University of Wollongong, New South Wales, Australia; 14 Australian Health Services Research Institute, University of Wollongong, New South Wales, Australia; 15 Department of Psychiatry, University of Melbourne, Melbourne, Australia; 16 ISN Innovations, Institute for Social Neuroscience, Melbourne, Australia; 17 School of Computing and Information Systems, University of Melbourne, Melbourne, Australia

**Keywords:** Evidence synthesis, Dementia, Older adults, Cognitive interventions

## Abstract

Systematic reviews and meta-analyses are critical in health-related decision-making, and are considered the gold standard in research synthesis methods. However, with new trials being regularly published and with the development of increasingly rigorous standards of data synthesis, systematic reviews often require much expertise and long periods of time to be completed. Automation of some of the steps of evidence synthesis productions is a promising improvement in the field, capable of reducing the time and costs associated with the process.

This article describes the development and main characteristics of a novel online repository of cognitive intervention studies entitled Cognitive Treatments Article Library and Evaluation (CogTale). The platform is currently in a Beta Release phase, as it is still under development. However, it already contains over 70 studies, and the CogTale team is continuously coding and uploading new studies into the repository. Key features include advanced search options, the capability to generate meta-analyses, and an up-to-date display of relevant published studies.

**Supplementary Information:**

The online version contains supplementary material available at 10.1186/s13643-021-01787-2.

## Background

Dementia is one of the world’s most pressing health challenges, affecting around 50 million people globally, with projections that by 2050 this number will increase to around 152 million [1]. Reflecting this challenge, research into the development of pharmacological and non-pharmacological treatments to prevent, delay, or slow down the progression of dementia has accelerated at an unprecedented rate over the past two decades [2, 3].

Cognition-oriented treatments (COTs) are one promising class of non-drug treatment focused on engagement in a range of structured and unstructured activities to maintain or improve cognitive and daily function [4–6]. Interest in the potential of COTs to improve outcomes for older people with or at risk of dementia is reflected in an ever-growing number of clinical trials and evidence-based synthesis efforts in the field of COTs. However, due in part to the heterogeneous nature of many of these interventions, and the varying methodological quality of trials and reviews [7], the resulting body of literature has been inconsistent. Furthermore, clinical translation for quality research and subsequent outcome results has generally been slow.

Systematic reviews and meta-analyses are widely regarded as the “gold standard” in evidence-based synthesis efforts that affect health-related decision-making [8]. Unfortunately, the proliferation of trials, coupled with increasing complexity and rigor in the conduct of systematic reviews, results in an unacceptably slow evidence synthesis pipeline. Consequently, with rigorous reviews often taking 2 years or more to achieve publication, new relevant trials published in the interim are not able to be included, which can, in turn, lead to biased conclusions or duplication of trials.

Building on previous efforts to create trial archives in the areas of psychological therapies following brain injury (e.g., PsycBite) [9], and rehabilitation treatments more broadly (e.g., PEDro) [10], we developed the Cognition-oriented Treatments Article Library and Evaluation (CogTale) [11], an online COT trial repository and semi-automated platform for evaluation of trial quality, continuous rapid synthesis, and dissemination of evidence. Although the CogTale platform incorporates some functionality included in such meta-analytic software as Cochrane RevMan [12] or Meta-Essentials [13], it also offers other features as further described below that make it a comprehensive platform different from anything currently available. We believe that CogTale is a platform with a potential to impact research, clinical practice, and public information. In this paper, we briefly describe the main methodologies and features of the platform, its status and future directions.

## Methods

### Overview

CogTale forms an element in the research roadmap of the Cognitive Interventions Design, Evaluation, and Reporting (CIDER) group, an international team dedicated to advancing the field of COT research in the older adult population [14–16]. Key objectives of CIDER are promoting the methodological rigor of interventions and trials, acceleration of evidence synthesis, and dissemination of reliable and responsible information to the general public, researchers, and clinicians.

The Melbourne eResearch Group (MeG) [17] implemented the CogTale platform, which offers a combination of both public and restricted-access services. The restricted-access section is a custom-built web application supporting a comprehensive pipeline from data entry to displayed outputs.

We established CogTale as a three-tier web application, with a single-page react-based user interface. The backend is based on a NodeJS application that provides a representational state transfer (REST)-based application programming interface (API) supporting job execution, messaging, and other business management services. The analysis sub-module, implemented in R, supports statistical analyses and report generation. Record and file data are persisted to a MongoDB database. Together, the three-tiered platform supports the identification and queueing of targeted articles, data extraction, data analysis and reporting, quality assurance, and administration of the entire review process. The application is integrated with a WordPress-based public-facing website.

### Application inputs

Eligible studies for inclusion on the CogTale platform are articles reporting controlled trials of cognition-oriented interventions targeting older people on the continuum of cognitive health and impairment, ranging from cognitively unimpaired to people with a formal diagnosis of dementia.

Currently, the search for eligible articles is being performed manually, primarily through Google Scholar using terms related to COTs (e.g., “cognitive training,” “cognitive stimulation,” “cognitive rehabilitation,” “brain training”), cognitive status (e.g. “healthy,” “unimpaired,” “mild cognitive impairment,” “dementia”), and to aging (e.g. “older,” “elderly”). Studies are also added based on the expertise of the project team with previously published work in the area. Both administrators and coders can search for studies and add them to the platform, and platform users can suggest a study be included by using the *Contact us* function. Although not yet functional, an automatic article search feature (“discovery”) is currently under development. Once active, this feature will enable retrieving studies from selected bibliographic databases following related search terms and shortlisting them to the database on an ongoing basis. This function works by querying the databases monthly. The content of sites and their respective search APIs are substantially different. Consequently, the search strategy has been customized for each site, with queries designed to return recent publications of relevance to CogTale. The backend keeps track of the unique identifiers for each publication so that it knows what is new, what has been selected or rejected for review. An editor reviews the discovered publications list, and tags each item to be reviewed or ignored.

Trained coders extract detailed design and methodological data from each eligible trial, guided by a coder manual, into a data entry form that also includes all relevant means and standard deviations for every measure, condition, and time-point in a trial. We organized items on the data extraction form based on the following study aspects:

- Methodological/design data
  - Setting and design of the study
  - Primary and secondary outcomes
  - Interventions: nature and dose
  - Intervention targets and components (experimental and control)
  - Populations and sub-populations included
  - Measures used
  - Statistical analyses done
- Numerical data/findings
  - Sample size, means, and standard deviations in relation to each measure, condition, and time point reported in the study

We designed the coder dashboard such that half the screen includes the data extraction form while the other half includes several possible tabs between which coders can toggle, namely, a portable document format (PDF) viewer of articles related to the trial being coded, the analysis being conducted on the data (see below), a discussion panel, and the coding manual.

**Fig. 1:**
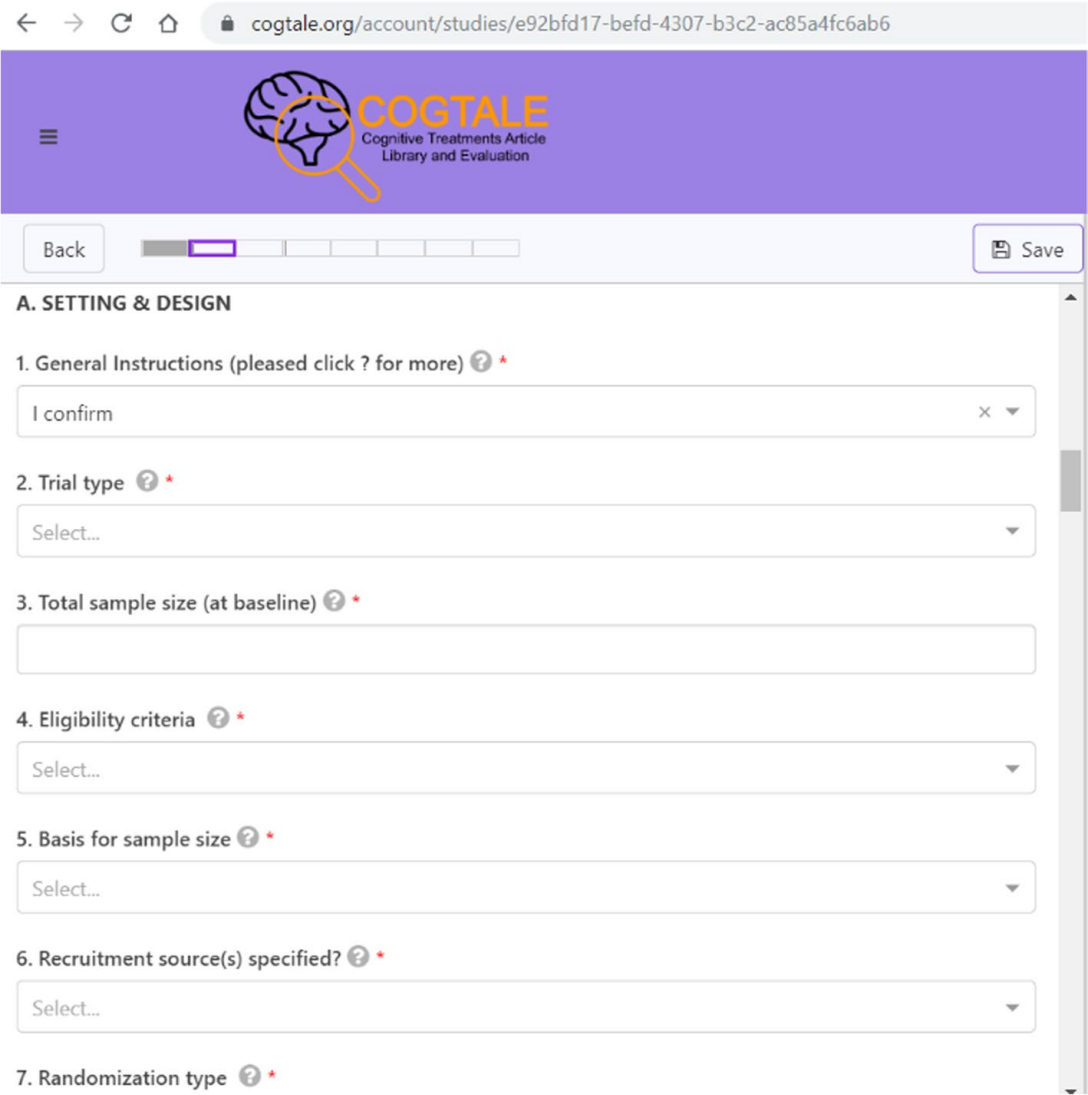
Data extraction items

For each data extraction item, coders can choose from several response alternatives (termed “vocabularies”), with some options being mutually exclusive and some allowing multiple vocabularies. Coders also have the option of adding an additional response if deemed appropriate. This is automatically added to the list of vocabularies for that item. A sample subset of data extraction items from one section is shown in Fig. 1.

Upon completion of the data extraction process, coders are required to write a plain language summary highlighting the main findings, strengths, and limitations of the study.

There are 5 possible study statuses: *queued* (before coding has commenced), *in progress* (when the study has been assigned to a coder), *requirements met* (when all required sections have been completed by the coder), *revisions required* (when an administrator has reviewed the coding and has decided that it is incomplete or needs to be revised), and *verified* (when an administrator has approved the coding of the study). This ensures that data extraction undergoes systematic monitoring and evaluation by research personnel with appropriate expertise.

Data extraction for each study is conducted by one coder and reviewed by an administrator. A second administrator performs data checks regularly to further ensure that studies are coded accurately. When a study is coded by an administrator, it is reviewed by another administrator before it is verified.

### Application processing/algorithms

#### Methodological quality indices

The detailed data extracted from each trial is used to calculate three common indices reflecting the methodological quality of the study, namely: the PEDro scale [18], the Jadad rating [19] and the Cochrane Risk of Bias tool [20]. In relation to these scales, the data extracted is used to determine the score of each item included in each of the scales, such as blinding of participants and outcome assessors, the method of randomization, and retention rates, amongst other factors. Not every item on the calculated methodological quality index is necessarily represented by a single item on the data extraction form, and at times a combination of responses is used to determine the score on a particular item. We developed and refined algorithms describing these scoring rules in an iterative way to achieve close agreement with manual scoring of the above indices (available from the authors upon request).

#### Effect estimates and confidence intervals

Once the “Results” section of the data extraction form is complete, estimates of treatment effect (Hedges’ *g*) [21] along with their confidence intervals are automatically calculated for each measure and time point. The treatment effect estimates are based on the standardized mean differences between experimental and control groups. For all outcomes, a positive effect favors the experimental condition, whereas a negative effect favors the control condition.

Where a study has only reported data for independent subgroups, a combined effect size across the subgroups is calculated by conducting a fixed-effect meta-analysis on the subgroups for that study [22]. In addition, for studies comprising more than one control and/or experimental groups, pairwise comparisons are conducted between the control and experimental group(s) and/or between experimental groups. The corresponding standard errors are adjusted using the method of Rucker et al. [23]. The effect estimates and adjusted standard errors are then pooled using a fixed-effect meta-analysis.

#### Meta-analysis

The treatment effect estimates from multiple studies in the platform can be synthesized through a quantitative meta-analysis carried out according to the users’ criteria.

Based on recent recommendations [24], a pooled Hedges’ *g* is calculated for each outcome using the random-effects model with the restricted maximum likelihood (REML) heterogeneity estimator (τ^2^) in conjunction with the Hartung–Knapp–Sidik–Jonkman (HKSJ) [25, 26] method to calculate the corresponding confidence intervals. The REML estimator outperforms other heterogeneity estimators and the HKSJ correction is not influenced by the magnitude or estimator of τ^2^ and it is insensitive to the number of studies [24].

Heterogeneity in effect estimates across studies is tested using the *Q*-statistic (with *p* < 0.10 indicating significant heterogeneity) and its magnitude is quantified using the *I*^2^ statistic, which is an index that describes the proportion of total variation in study effect size estimates due to heterogeneity. This is independent of the number of studies included in the meta-analysis and the metric of effect sizes [27]. As the *Q*-statistic has low power when the number of studies is small [28], 95% prediction intervals are calculated to quantify the extent of heterogeneity in the distribution of effect sizes [29]. The prediction interval is an estimation of the range within which 95% of the true effect sizes are expected to fall.

If the meta-analysis contains a study with multiple treatment effects for related outcomes for the same participants (e.g., two different measures of depression), a summary effect estimate is computed by combining the data from all the related outcomes. However, this approach requires that a correlation coefficient be specified for these calculates. Since this will vary between different outcome domains, the analyses default to a correlation of Pearson’s *r* = 0.50 [22].

#### Grading of the evidence

In relation to each outcome included in the quantitative meta-analysis, the platform automatically calculates a measure of certainty in the evidence. The algorithms used for calculations of certainty include mean methodological quality of included studies, heterogeneity between studies, and precision of the effect estimate (number of participants). The certainty is calculated as being either “low”, “moderate”, or “high” with regard to each outcome. An additional file provides further information about the grading of the evidence (see Additional file 1).

#### Evidence summaries

In relation to each outcome included in the meta-analysis, the platform further generates an evidence summary. The algorithm rules used for generating these summaries include the magnitude of the effect relative to that outcome, the statistical significance of the effect, and the certainty in the evidence. The platform makes modest or strong recommendations in favor or against a treatment approach, population, and outcome when the certainty in the findings is moderate or high, respectively. It makes no recommendations for evidence rated as being of “low” certainty.

### User interface

The website contains several general features (About, Resources, News, Twitter feed, study and report metrics, etc.). The website landing page is also the main gate to accessing the application.

**Fig. 2:**
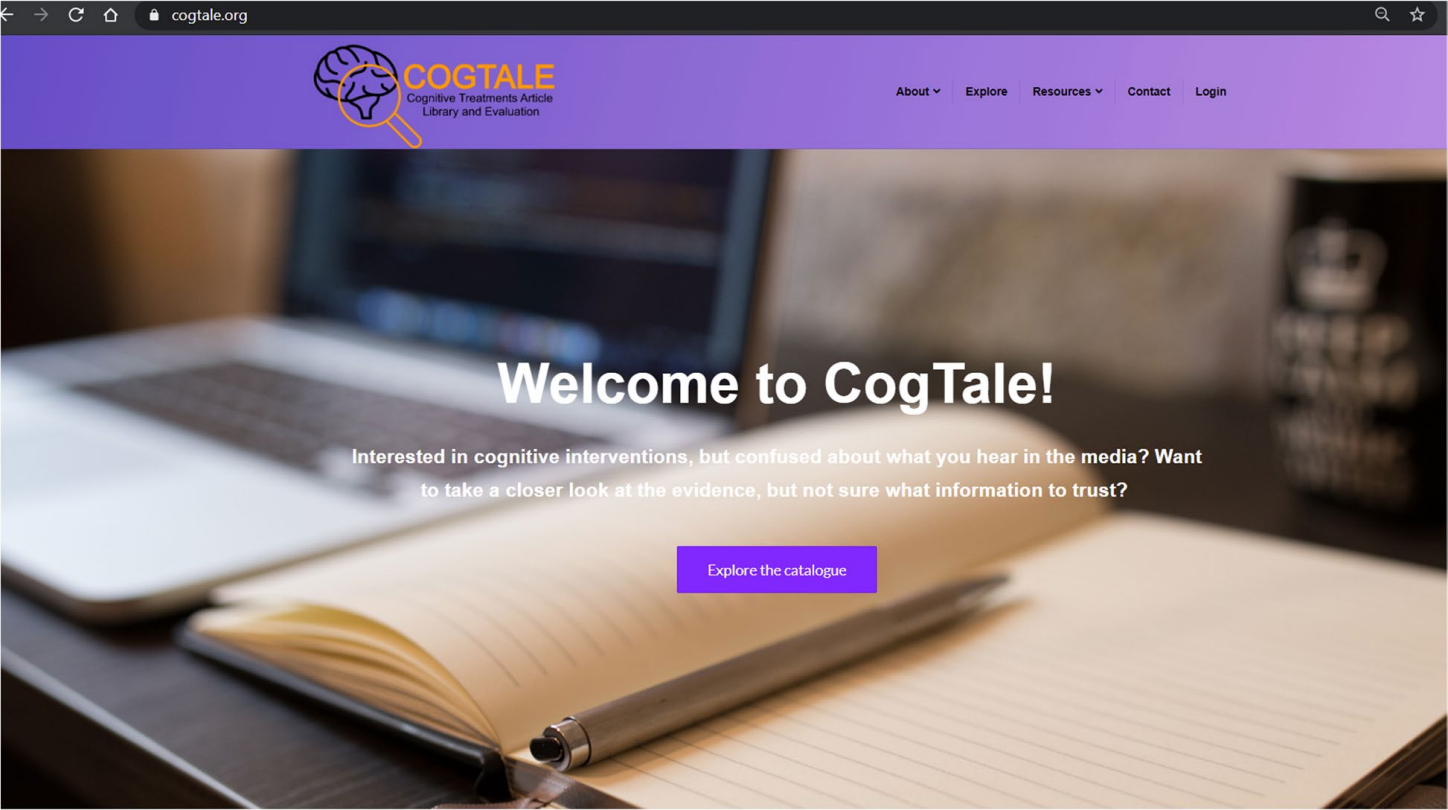
Landing page

A “Login” function permits the creation of an account or to log in to an existing one. Once logged in, users can manage information on their profile and access the “Explore” function from the user menu, from where they can browse all studies stored on the database. A section of the landing page is displayed on Fig. 2.

To date, we specified four account types, each with different permissions, and all free of charge.

A “General User” account is a general profile, available for anyone who wishes to bookmark or export studies, or to conduct meta-analysis procedures and receive associated reports.

Users classified with “coder A” accounts have permission to extract all relevant trial data into the CogTale platform but they cannot self-assign studies. An administrator must assign these. Further, questions in the data extraction form that require greater judgement or specialist knowledge may be disabled for this user type and a more experienced coder (i.e., a coder B or administrator) must complete them. “Coder B” accounts can add new studies, self-assign studies for coding, and extract all relevant data in the question set. Finally, “Administrator” accounts grant permission for users to establish coding accounts as well as perform any of the tasks mentioned thus far. Following a review process, Administrators can also change the status of a study into “verified.” They are also able to add or change questions and response alternatives in the data extraction form. Thus, the CogTale data extraction function is flexible and can be adapted to changes in research conditions or attributes that may be reported.

### Application functions

Below, we summarize the main application-related functions available within the user interface:

#### Search

Users can browse or perform searches to locate specific studies or groups of studies in the database.

Users may simply browse the catalog by navigating through the list or by writing the name of the author and the title or the journal in which the study was published. The available studies may be viewed either as a list or in tabular form, where users can sort them on several aspects, including author name, year of publication, trial design (e.g., randomized controlled trial), population of interest (e.g., dementia), or intervention (e.g., cognitive training). Two sliding bars further allow users to filter studies based on a selected range of publication years and methodological quality scores.

Advanced search options permit users to specify numerous other search properties, broadly corresponding to all main sections in the data extraction form, such as the delivery format or setting of the intervention, the type of control group, or the sample size of a study, amongst other properties.

Users can export the results retrieved through the search by clicking on the “Export” function, where tab-separated values (TSV) format allows transfer of the information from the database to a spreadsheet. Citation information can also be downloaded in research information system (RIS) format for import into reference/citation manager applications, like Zotero, Citavi, Mendeley, and EndNote.

#### Single study results page

By clicking on a given study, users can view detailed information about it. The single study results page displays the status of the study in the pipeline, which (as noted above) can vary from being “in progress” to being “verified”, depending on its stage in the coding process. For each study in the database, the single study results page displays citation information and the abstract, an expandable table of methodological quality scores (item level and total), effect size tables (for each measure, time points, and populations) and a plain language commentary or summary provided by the coder.

#### Meta-analysis wizard

Users can select multiple studies and submit them to a meta-analysis by clicking on the “analyze” button, which will in turn bring up the meta-analysis wizard (MAW). The MAW allows users to define the scope of the meta-analysis by specifying populations (e.g., people with mild cognitive impairment), targets (e.g., study participants, caregivers, or clinicians), and broad as well as specific outcomes of interest (e.g., global cognition, delayed recall).

**Fig. 3:**
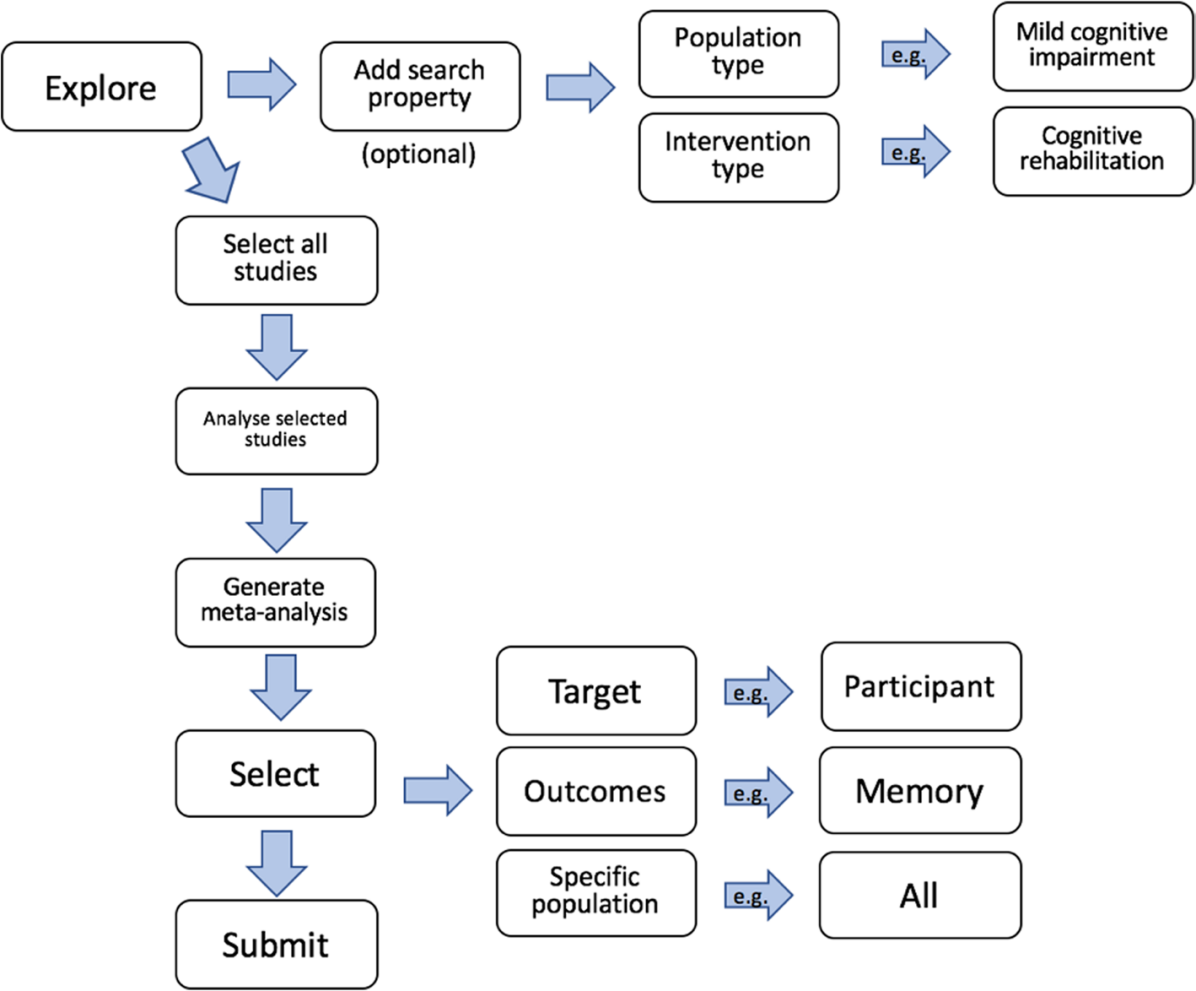
Meta-analysis wizard

Only studies that include relevant data (i.e. means and standard deviations for each group) can be pooled together in a meta-analysis. Therefore, if a study is retrieved by the search but it does not have the data required to allow computation of effect sizes, CogTale automatically excludes it from the analysis. For any given outcome or population of interest, data can be pooled if there are at least 3 studies that provide data for that effect estimate. By default, all available outcomes and populations that meet the minimum criteria are selected for meta-analysis. However, users can customize the search to remove any outcome and any population from the analyses if desired. Figure 3 displays the process of searching for studies and generating a meta-analysis.

### Application outputs

#### Single study results page

**Fig. 4:**
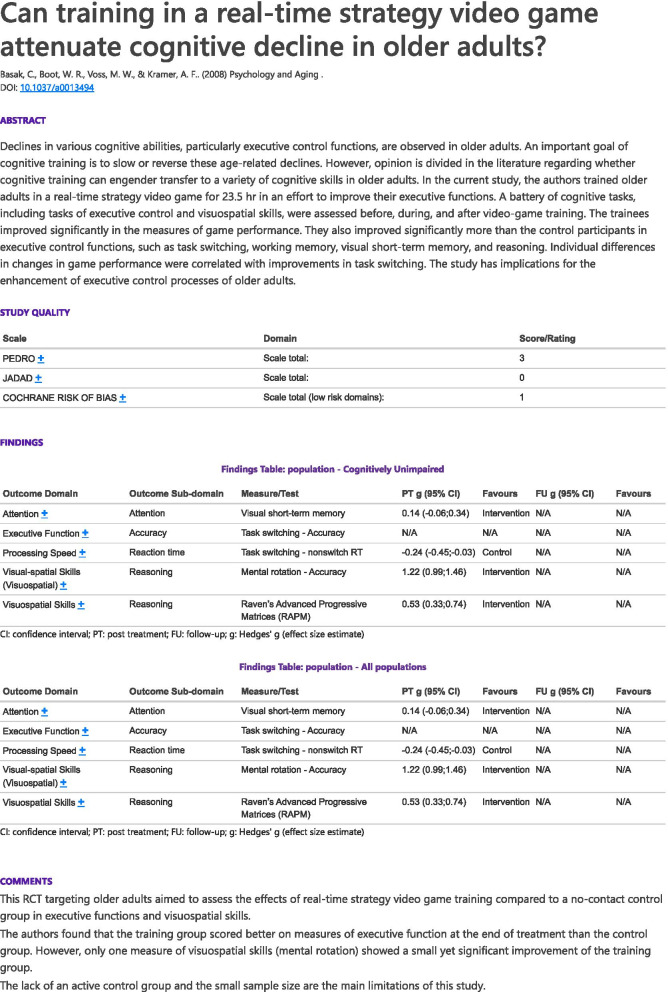
Example of single study results page

Figure 4 displays an example of the single study results page, which can be viewed, saved, or printed. All tables in the results page are expandable and allow users to see more detailed information.

#### Meta-analysis report

**Fig. 5:**
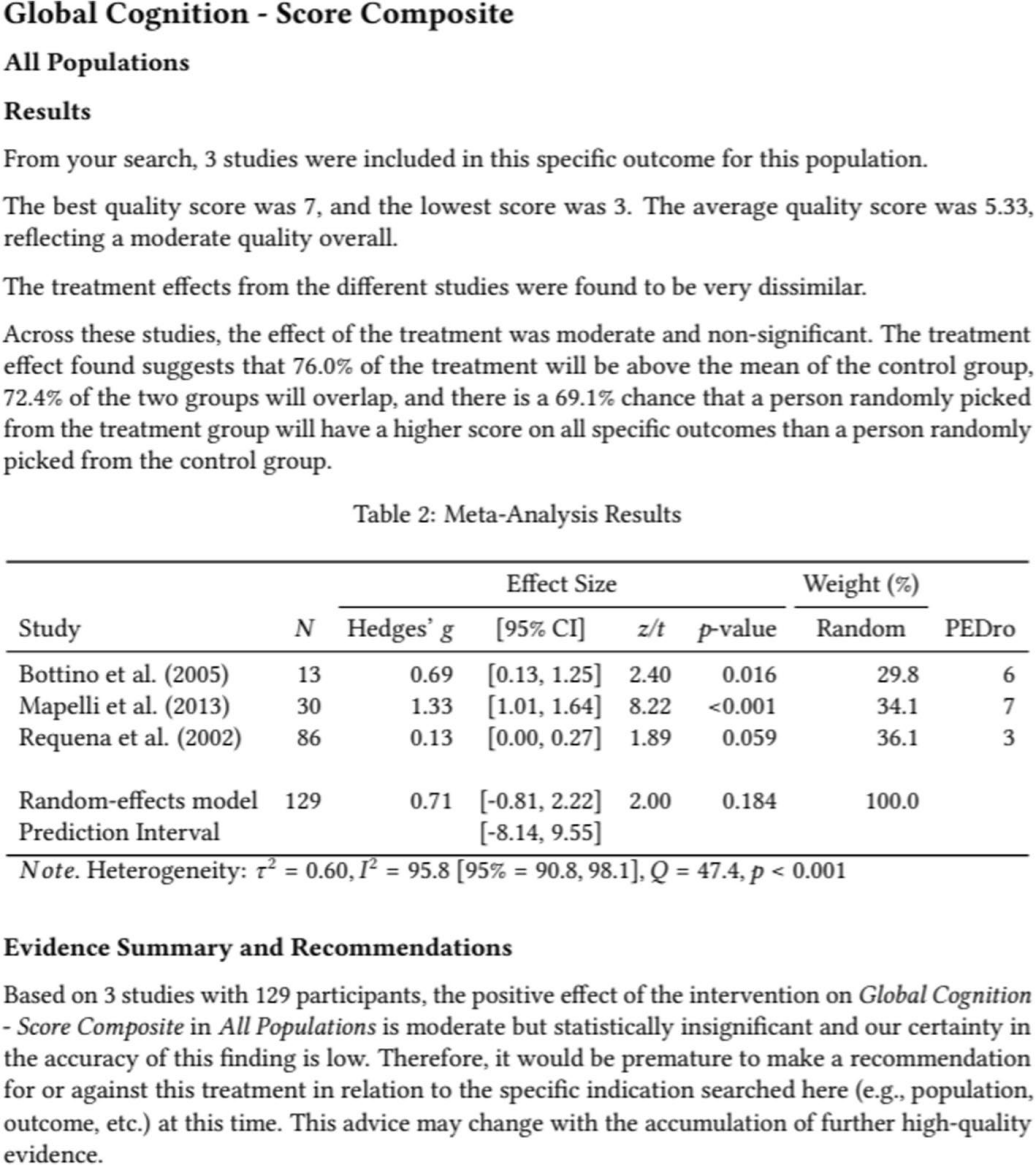
Data synthesis report: example of results

**Fig. 6:**
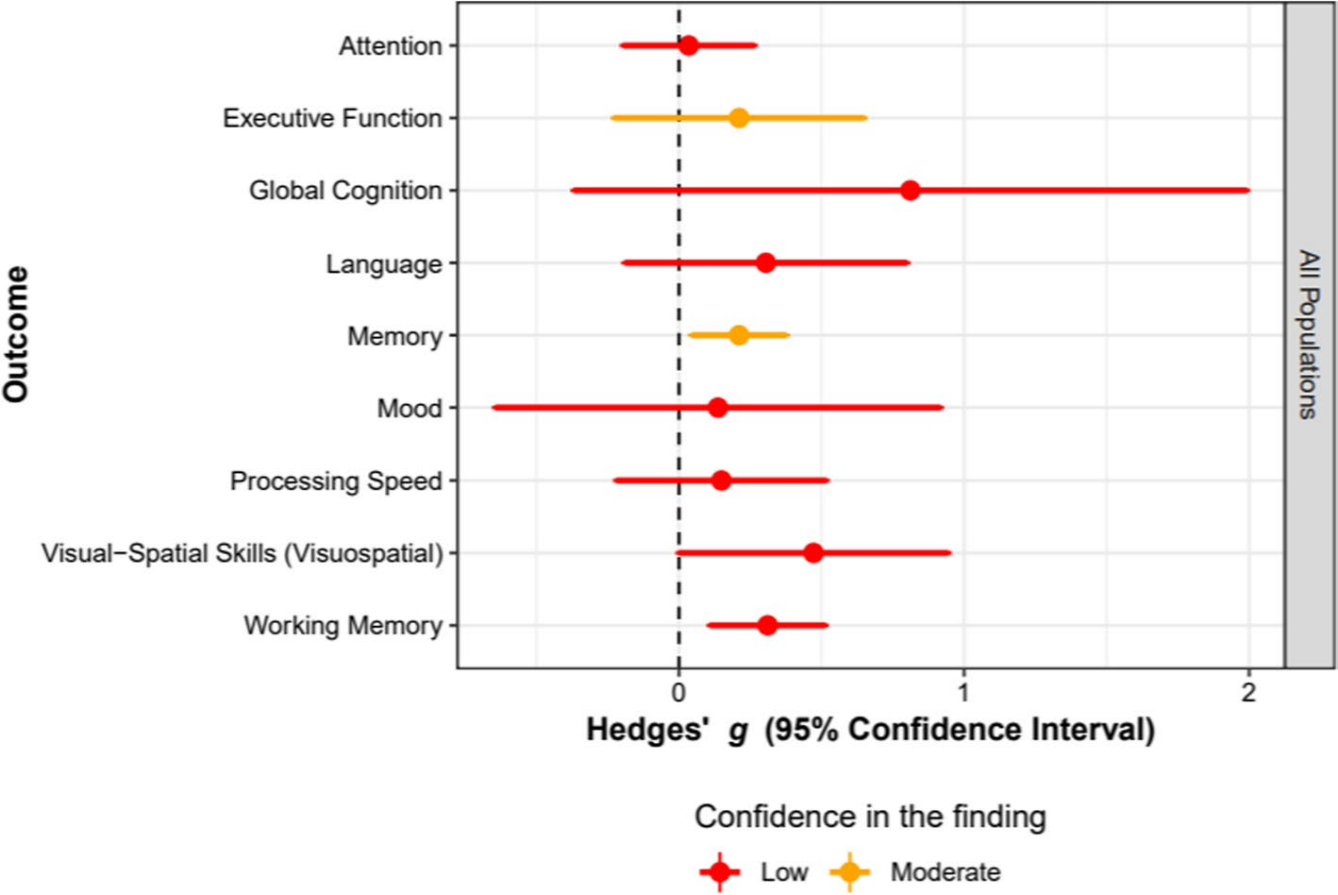
Example of data synthesis report: confidence in the finding chart

Once a meta-analysis is specified and submitted, users receive a comprehensive report containing tables, figures, and results summaries as an email attachment. Table 1 summarizes the sections included in a report. Figure 5 presents an example of a section from the report, and Fig. 6 presents an example of the figure included in a report, showing the relation to each outcome, the effect estimate and confidence interval together with the certainty of the finding.

**Table 1:** Data synthesis report: sections

Section	Content
Overview	An overview of cognition-oriented treatments
Disclaimer	A message to remind the user that www.cogtale.org is still under development with studies being routinely entered and, thus, results need to be interpreted with caution
Search results	A summary of the focus of the search and the number of outcomes included in the meta-analysis
Overall summary	An explanation of how results are interpreted, a summary of results and recommendations for each domain and each population, and a summary plot showing the effect sizes, confidence intervals, and confidence in the finding
Detailed report	Detailed information about the results for each outcome and population group, including forest plots
References	List of articles reporting studies included in the meta-analysis
Statistical information	A link to a section of www.cogtale.org with information about the meta-analytic process

The pooled effect size is interpreted in-text using three different methods. Firstly, the measure of non-overlap (*U*_3_) [30] gives the percentage of cases from the experimental condition that exceeds the mean of the control condition. Secondly, the percentage of overlap between the control and experimental conditions is reported [31]. Lastly, the probability of superiority (also known as the common language effect size) [32] which gives the probability that a person picked at random from the experimental condition will have a higher score than a person picked at random from the control condition. For example, an effect size of Hedges’ *g* = 0.50 would be presented in the report as the following:

*The treatment effect found suggests that 69% of the treatment will be above the mean of the control group, 80% of the two groups will overlap, and there is a 64% chance that a person randomly picked from the treatment group will have a higher score on all specific outcomes than a person randomly picked from the control group*.

## Results

CogTale is currently in a Beta Release phase and, as such, it is still under development. We are continuously updating the platform and adding new studies regularly to the repository. Currently, approximately 70 trials are in the database, most of which have data required to be included in a meta-analysis. There is currently a total of 67 user accounts, including 35 General Users, 7 Coder A, 13 Coder B, and 12 Administrator accounts, all of which represent researchers from different countries. To date, 350 meta-analysis reports have been generated and e-mailed. We anticipate these figures will grow substantially as data are added to the database, and with additional coders being added to the team.

Preliminary evidence, based on a small number of inter-rater reliability checks, suggests that the data extraction process is reliable overall and that data extraction by novice coders is only minimally different from coding by more experienced coders, and discrepancies are generally easily resolved following supervision. A “Coder Manual” currently assists coders and, based on our experience and feedback received to date, we are in the process of revising and expanding this manual with clarified coding guidelines. We will also add instructional videos to the platform in the coming months. We are in the process of developing several education and training workshops and webinars directed at prospective coders, and researchers interested in meta-analysis more generally.

We are also working towards revising our approach to the classification of measures and outcomes. The relative lack of consensus on the most appropriate ways to classify cognitive and other measures in psychosocial research is well recognized and our team of international leaders in cognitive science and neuropsychology are working to establish a both pragmatic and theoretically-informed approach to the classification of measures and outcomes.

Additionally, the platform currently only allows for means and standard deviations to be entered while coding a study, but we expect to be able to enter other types of measurement (e.g., mean change, standard error) in as the database grows.

CogTale presently includes only trials of COTs in the older adult population. However, plans are underway to broaden the scope of the database to other populations and interventions. Our team is currently trialling several approaches to the triaging of studies for inclusion in the database, and we expect that the process may follow a combination of pre-defined and adaptive approaches to accommodate specific trends, projects, and data analytic plans.

Finally, a novel feature currently under development is the addition of a series of regularly updated “Citizen Briefings” which will be short summaries in non-technical language of the evidence for the effect of specific types of intervention on specific populations, e.g., the effects of cognitive rehabilitation on quality of life of people with dementia. These reports will be available on the website for anyone to read and will be continuously updated with the inclusion of newer studies. We believe that this feature will be of great value for a range of stakeholders interested in the evidence on the effectiveness of various forms of treatment for various populations.

## Conclusions

This paper has reviewed the development and main characteristics of CogTale. This novel platform has been successfully launched and, as mentioned, is a promising offering capable of filling a significant gap in the field of meta-analysis. We are confident that the features of the platform and the wide range of levels of complexity and rigor provided for meta-analysis, make CogTale an innovative and unique solution aimed at serving not only researchers and clinicians, but also non-expert members of the population hoping to keep up to date with what the evidence demonstrates.

## Supplementary Information

**Additional file 1.** Description of data: Information about the grading of the evidence.

## Data Availability

The data and materials presented in this article are available from https://www.cogtale.org. The source code or algorithms are not publicly accessible but are available on request subject to distribution constraints.

## Abbreviations

API: Application programming interface
CIDER: Cognitive Interventions Design, Evaluation, and Reporting
CogTale: Cognitive Treatments Article Library and Evaluation
COT: Cognition-oriented treatments
HKSJ: Hartung-Knapp-Sidik-Jonkman
MAW: Meta-analysis wizard
MeG: Melbourne eResearch Group
PDF: Portable document format
REML: Restricted maximum likelihood
REST: Representational state transfer
RIS: Research Information System
TSV: Tab-separated values
