# Supplementary material for: CogTale: an online platform for the evaluation, synthesis, and dissemination of evidence from cognitive interventions studies
Source: Syst Rev. 2021 Aug 24;10:236. doi: 10.1186/s13643-021-01787-2 (PMC8383388; doi:10.1186/s13643-021-01787-2)
Supplement: Supplementary file 1 — Additional file 1. Description of data: Information about the grading of the evidence. [file 13643_2021_1787_MOESM1_ESM.docx]

Appendix

*As written in the paper…..*

*“….the certainty in the evidence. The platform makes modest or strong recommendations in favour or against a treatment approach, population and outcome when the certainty in the findings is moderate or high, respectively. It makes no recommendations for evidence rated as being of ‘low’ certainty….” – direct reader to Appendix for more detail here…*

The certainty in the accuracy of the estimate and recommendations

CogTale Reports are generated once a meta-analysis is specified and submitted. For each outcome these reports summarise a pooled effect size, confidence interval, heterogeneity, and the statistical significance of the outcome. A plain language summary of each finding also gives an index of the *certainty in the accuracy* of a meta-analysis finding, that is, an estimate of how confident we can of the result.

The certainty in the accuracy of the estimate is determined by a unique algorithm based on several factors. These include a combination of; a) the heterogeneity of the effect (e.g., the I^2^ statistic), b) the methodological quality scores of the synthesised studies (e.g., the PEDro score), and c) the combined sample size. Findings associated with low confidence are likely to change with the addition of more relevant and high-quality studies, whereas findings associated with high confidence are more likely to remain the same if new studies were added to the analysis.

Based on a combination of the effect size, quality rating, and certainty in the accuracy of the finding, a recommendation may also be included in the plain language summary of the meta-analysis result. For example, where the overall quality of the included studies is high, and the certainty in the accuracy of the finding is moderate to high, a recommendation may be made as to the veracity of the intervention effect. The recommendation could indicate the treatment is unlikely to be effective, or an intervention could be recommended with a high degree of confidence.

Where there is low confidence in the accuracy of the estimate, no recommendation is made for or against the intervention. However, a caveat that the advice may change with the accumulation of further high-quality evidence is included.
